# Optical Sensors Based on Plastic Fibers

**DOI:** 10.3390/s120912184

**Published:** 2012-09-05

**Authors:** Lúcia Bilro, Nélia Alberto, João L. Pinto, Rogério Nogueira

**Affiliations:** 1 Instituto de Telecomunicações—Pólo de Aveiro, Campus Universitário de Santiago, 3810-193 Aveiro, Portugal; E-Mails: nelia@ua.pt (N.A.); rnogueira@av.it.pt (R.N.); 2 Polytechnic Institute of Viana do Castelo, Avenida do Atlântico, 4900-348 Viana do Castelo, Portugal; 3 Centre for Mechanical Technology and Automation, Department of Mechanical Engineering, University of Aveiro, Campus Universitário de Santiago, 3810-193 Aveiro, Portugal; 4 Department of Physics & I3N, University of Aveiro, Campus Universitário de Santiago, 3810-193 Aveiro, Portugal; E-Mail: jlp@ua.pt

**Keywords:** plastic optical fiber (POF), sensors, structural health monitoring, medicine, environment, biological and chemical area

## Abstract

The recent advances of polymer technology allowed the introduction of plastic optical fiber in sensor design. The advantages of optical metrology with plastic optical fiber have attracted the attention of the scientific community, as they allow the development of low-cost or cost competitive systems compared with conventional technologies. In this paper, the current state of the art of plastic optical fiber technology will be reviewed, namely its main characteristics and sensing advantages. Several measurement techniques will be described, with a strong focus on interrogation approaches based on intensity variation in transmission and reflection. The potential applications involving structural health monitoring, medicine, environment and the biological and chemical area are also presented.

## 1. Introduction

The early studies on optical fiber technology-based sensors were published in the 70s and related to the first medical and industrial fiber optic endoscopes [1]. Up to now, there have been a growing number of research groups dedicated to the exploration of this technology. Studies followed towards the development of new optical fiber based sensors, for a wide variety of applications, overcoming the difficulties inherent to the measurement of a parameter, where traditional systems are not appropriate. Optical fiber sensors have several advantageous features: they are compact, lightweight and enable the implementation of multiplexing schemes. As the principle of operation is based on an optical signal, they also exhibit immunity to electromagnetic interference. However, the expectations for the production of optical fiber sensors at low or competitive cost compared to the well-established conventional technologies are still demanding [2]. Plastic or polymer optical fiber (POF) can meet these expectations.

The term optical fiber is often synonymous with glass optical fiber (GOF), although chronologically, the first POF was produced by DuPont at the end of the 60s, so POF appeared at the same time as glass fibers. Nevertheless, GOF dominated the market since they presented lower attenuation and POF was set aside. Due to the incomplete purification of the monomers used in the polymerization reaction, the POF attenuation remained at 1,000 dB/km. Thereafter, the attenuation was reduced to 125 dB/km (650 nm). Comparatively, GOF presented attenuations in the order of 1 dB/km (1,300 nm or 1,500 nm) and were already available commercially at low prices [3]. An excellent historical perspective on developments in POF can be found in [4]. Recent progresses in polymer technology and applications, including the improvement of transparency of the materials, have nowadays led to POF being considered a viable alternative to the dominant technologies in the marketplace [5].

The European POF industry is one of the driving forces behind the development of POF technology, applications and standards. Several European consortia have been created for the development of new components, fiber assemblies and transmission techniques to enable high speed optical links. They are constituted by SME companies, non-profit research centers, universities and telecom operators [6,7]. Bayern Photonics also developed a project named POF-Atlas [8] in order to stabilish the guide product for polymer optical fibers and components. This project was supported by the German Federal Ministry of Education and Research and the Bavarian Ministry of Economic Affairs, Infrastructure, Transport and Technology, and technically implemented by the Polymer Optical Fiber Application Center (POF-AC). Moreover, the Plastic Optical Fiber Trade Organization (POFTO) actively promotes the proliferation of POF systems, directed to both data and non-data communication markets. It is responsible for the POF Symposium at the OFC/NFOEC conference organization. The POF scientific community has been pressing for a relevant event and as a result the International Conference of Plastic Optical Fiber (ICPOF) has been established since 1992.

In this paper, a brief review of POF sensors and their applications is presented. The plastic fiber technology is summarized and several sensing mechanisms are described. This paper doesn't aim to present a thorough review of all POF sensor approaches, but rather to focus on more simple and low-cost interrogation approaches based on intensity variation measurement techniques in transmission and reflection. Some brief considerations about other sensing techniques such as interferometry and fiber Bragg gratings (FBGs) will also be made. The applications of POF sensors in the areas of structural health monitoring, medicine, environment and biology and chemistry will be described.

## 2. POF Technology

POFs have the intrinsic advantages of any optical fiber and in addition are easy to handle and flexible. Due to their large core diameters (typically 0.25 mm–1 mm), POFs allow the use of low precision connectors which reduces the total cost associated with a complete system. The Young's modulus value of bulk poly(methyl methacrylate) (PMMA) is 3.2 GPa while for silica fibers it is 72 GPa [9]. Moreover, this polymer is also characterized by being resistant to impacts and vibrations and having lower density (1,195 kg.m^−3^) and higher elastic deformation limits (10%) [9]. For POF, the mechanical strength depends on the composition, drawing process, presence of dopants and geometry [10]. Peters *et al.* [11] summarized the measured tensile properties for single mode PMMA POF and the Young's modulus changed from 1.6 GPa to 5.0 GPa. The fracture strain was around 30% for strain rates between 0.01 and 3.05 min^−1^. By selecting the optimum heat-drawing conditions, Ishigure *et al.* [10] presented a doped PMMA graded index (GI) POF with high elongation at break (>50%) and low length shrinkage (<1%). Compared with GOF, plastic fiber nevertheless also presents some drawbacks. It has greater attenuation coefficients, limited production, lack of standardization, few suppliers and the inability to operate at high temperatures, as the operating temperature limit for a POF ranges from 80 °C to 100 °C. Above these values, POFs begin to lose their rigidity and transparency. However, the temperature limit may be enhanced to 125 °C–135 °C with the use of a modified polyethylene or an elastomer with polyolefin coating [12–14]. The thermo-optic coefficient is negative, creating possibilities in the development of new techniques for the compensation of the temperature effect in deformation sensors.

Commercially, there are various optical fibers available that can be classified according to different criteria, such as the refractive index distribution, number of propagating modes, material composition and number of cores. The most often used material in the production of POF is the thermoplastic polymer PMMA, commonly known as Plexiglas^®^.

Regarding its optical transparency, the material is in its amorphous state. The typical refractive index for PMMA is 1.492. At room temperature and in an atmosphere with 50% relative humidity, the material can absorb up to 1.5% water. As shown in [15], the vibrational absorption presents a maximum at 620 nm and it is related to the sixth harmonic of the vibration of CH bond, with an attenuation coefficient of 440 dB/km. The transmission windows are located at wavelengths of 530 nm, 570 nm and 650 nm. One method that can be used to reduce the attenuation of the material is the replacement of hydrogen by greater atomic mass atoms, such as deuterium and fluoride. The first step-index (SI) POF with deuterium was produced by DuPont, in 1977 [16]. In 1982, the attenuation was 20 dB/km at 680 nm [17]. However, the disadvantage of this technique is that the constant presence of water vapor in the atmosphere will lead to the slow replacement of the deuterium by hydrogen atoms. Furthermore, the fluoride atom is heavier, leading to the displacement of the absorption bands towards the infrared. Perfluorinated (PF) POF is considered feasible for wavelengths from 650 nm to 1,300 nm, with attenuation coefficients lower than 50 dB/km [3]. However, the problem is to obtain a PF polymer which can be processed in its amorphous state. The PF polymers have one of the lowest refractive index of all the transparent plastics and, consequently, have been the preferred material for the coating.

**Figure 1.**
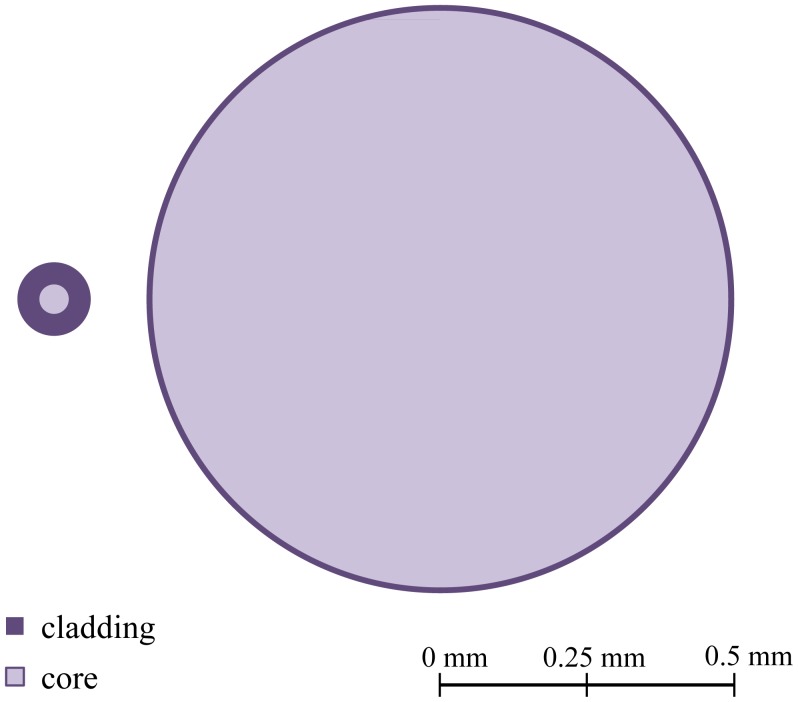
Comparison between the dimensions of MM-GOF and MM-POF.

Like GOF, the first developed POF had a SI profile. The POF has high values of numerical aperture (NA), typically 0.50, that translate into a very large number (10^4^–10^6^) of propagating modes. Figure 1 presents a schematic diagram of the NA and dimensions of a multimode (MM)-GOF and MM-POF. This high value facilitates the coupling of light in the fiber and reduces the losses associated with macrobending. Besides, it allows the analysis of the POF by geometric optics replacing the concept of modes for light rays. An important disadvantage is the greater modal dispersion and consequent lower bandwidth. The first POF with lower NA values, with a NA near 0.30, was presented by Mitsubishi Rayon [18] in 1995. Currently, all low NA POFs present, in fact, a double step index (DSI), which consists of two claddings surrounding the core.

The first single mode (SM) SI-POF was developed and presented by Koike *et al.* [19]. Compared to SM-GOF, the attenuation coefficients were very high (200 dB/km for 652 nm). Additionally, SM-SI-POF had lost the greatest advantages of the conventional POF: ease of handling and low-cost. Another type of SI-POF is formed by multi-cores (MC) and was first developed by AGC Asahi Chemical. In this type of fibers, multi-cores (up to about 200) are assembled during the manufacturing process, forming a POF with an overall diameter of 1 mm [20].

**Figure 2.**
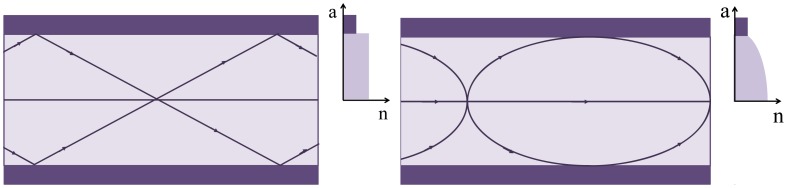
Refractive index profile and ray path (fundamental and highest order mode) for a SI (left) and GI (right) MM fiber.

In 2005, the first GI PMMA-POF, produced by the Korean company Optimedia Co., became commercially available. This type of fiber doesn't need cladding, but the process to obtain a gradual variation of the refractive index is complex. To date, the best results in the production of low attenuation POF with PF materials were obtained with cyclic optically transparent polymer (CYTOP^®^), developed by AGC Asahi Glass of Japan [21]. PF-GI-POF cable became available in the same year from Chromis Optical Fiber, which licensed the production of Lucina™ optical fibers from Asahi Glass. The attenuation coefficient was 10 dB/km for 1,300 nm [22]. Recently, also AGC Asahi Glass presented Fontex™, a GI-POF which has a double cladding structure that reduces the power losses associated with the curvature [23]. Figure 2 exemplifies a refractive index profile for a SI and GI optical fiber. In 2001, the first microstructured POF (mPOF) was presented, resulting from a collaboration between research groups from Australia and Korea. The mPOF has attracted the attention of the scientific community because it allows a custom control of the optical properties through the tailoring of the microstruture geometry. Kiriama Pty. Ltd., an Australian company formed in 2009, has exclusive access to commercialize this technology. Some of the plastic optical suppliers are summarized in Table 1.

## 3. Sensing Techniques

In the scientific literature, several sensing techniques used in the development of POF based sensors, are described, such as backscattering (optical time domain reflectometers, OTDR [24–26] and optical frequency domain reflectometers, OFDR [27,28]) and long period gratings [29]. Since most plastic optical sensors are based on intensity variation detection, a more detailed description will be presented for transmission, reflection, spectroscopic and evanescent field transduction mechanisms. A technique based in path difference will also be presented [30]. In addition, FBGs and interferometry will also be briefly described due to its increasing application.

### 3.1. Sensors Based on Intensity Variation Detection

Sensors based on intensity variation represent one of the first detection schemes used in optical fiber sensors. In terms of operating principle and instrumentation, it may be considered the simplest method. In general, experimental setups include a light source, the optical fiber and a photodetector or an optical spectrum analyzer. Commercially, several solutions for miniaturized solid-state sources and photodetectors are available, allowing the design and development of robust and portable acquisition systems. This is a suitable solution for engineering applications, where the accuracy in the power signal measuring is not critical or essential. The advantages of this measurement method are the ease of implementation, good price/quality ratio and simplicity in signal processing.

**Figure 3.**
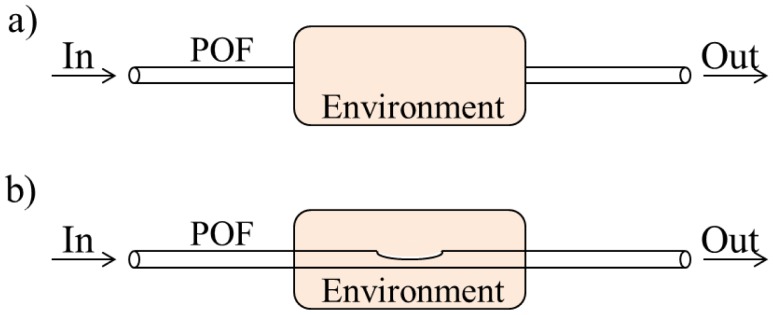
Schematic diagram of an (**a**) extrinsic and (**b**) intrinsic intensity-based sensor.

Despite the diversity of possible configurations, they can be classified into two broad classes, namely extrinsic and intrinsic sensors (examples are shown in Figure 3). The former is characterized by the use of the optical fiber as a means of optical signal transmission to an external environment. In the intrinsic scheme, the optical signal doesn't leave the optical fiber.

Another common classification relates to the type of optical signal acquisition. If the transmitter and receiver are in same or opposite ends it is considered a reflection or transmission configuration, respectively. An example of a transmission configuration is shown in Figure 3(a), where the intensity variation is related to the optical signal coupling between two optical fibers. This method was used by Kuang *et al.* [31] in the development of a strain sensor. Regarding to the reflection method, reflective surfaces are commonly used to reintroduce the optical signal into the fiber [32]. Other sensors are based on Fresnel reflection mechanisms [33,34]. In this case, special geometries at the end of the fiber have been used [35].

The various attenuation mechanisms include absorption, Rayleigh scattering and macro- and microbending radiation losses. Despite the efforts to minimize power losses, the dependence on these mechanisms is widely used in the development of micro- and macrobending based optical fiber sensors [36,37].

The problem of macrobending losses in optical fibers had received more theoretical emphasis. Boechat *et al.* [38] gave a considerable list of references on this matter. They developed the existing theory as a function of the properties of a large core multimode fiber (core diameter and NA) and the properties of the bend (radius and length). While properties of guided modes in an optical fiber can be described by modal methods, highly multimode fibers are often modeled using principles of geometrical optics. Durana *et al.* [39] studied the dependence of bending losses on POFs cladding thickness, through numerical results, using a ray-tracing model. Despite the obtained results, this approach is a time consuming procedure, since optical fibers with high NA allow nearly two million propagating rays. Each ray path, reflection point and new path is obtained using relative motion concepts.

**Figure 4.**
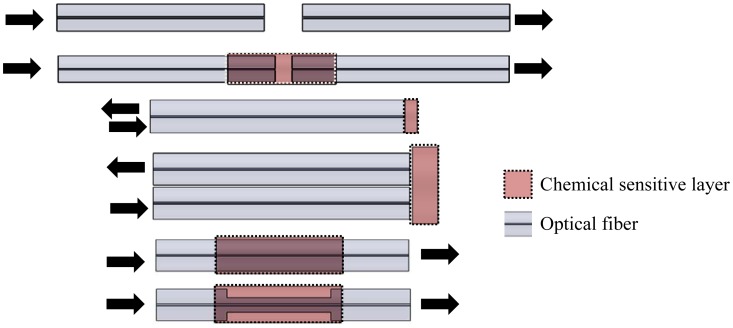
Schematic diagrams of several possible configurations of spectroscopic methods: (**a**)–(**d**) extrinsic and (**e**), (**f**) intrinsic configurations.

In the various reflection and transmission systems, there are several transduction mechanisms, including sensors based on spectroscopic methods and variations of the evanescent field. Spectroscopic detection has been employed in optical fiber sensors for chemical, biological and biochemical monitoring [40,41]. This method is based on absorption, fluorescence and refractive index changes. The optical power variation can be correlated with the concentration of the chemical or biological species [42]. As shown in Figure 4(a), in the case of direct spectroscopic measures, the sensors may be composed only by an optical fiber and cell samples. The attenuation due to the optical path cross can be related with the absorption properties or the medium scattering. Otherwise, chemical reagents can be immobilized on selective inorganic or organic matrices which are deposited onto the fiber [Figure 4(b), (e) and (f)] or on its end [Figure 4(c,d)]. An alternative of the method includes the use of fluorescent materials [41,43].

The optical signal propagates predominantly into the optical fiber core, however there is a small amount that penetrates into the fiber cladding and whose energy decays exponentially with the distance from the core (evanescent field). In standard optical fibers, the interaction between the evanescent field and the surrounding environment is negligible.

**Figure 5.**
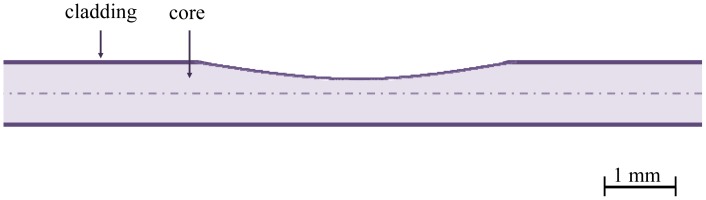
Schematic diagram of a side-polished fiber with core exposure.

The evanescent radiation power is dependent on the discontinuity in the core/cladding interface, the launch angle and the fiber dimensions. The methods used to increase the interaction between the evanescent field and environmental influences are side polishing (Figure 5), chemical etching, heat treatment and D-optical fibers. In 2008, a reliable method for inducing discontinuities based on a CO_2_ laser was published [44].

With reference to “imperfect” POF, two studies have addressed their modeling as curvature sensors [45,46]. Both considered a sawtooth shape sensitive area and the possibility of using air as external medium. Furthermore, the combined effect of macrobending and refractive index sensing in highly multimode unperturbed fibers had been reported [47]. An analytical analysis of a side-polished POF, considering these two different physical parameters was presented by Bilro *et al.* [48]. The developed model considered the geometry of the sensor, namely length and thickness, the angular distribution of the power and the possibility of multiple reflections in the side-polished section. A key point of the analytical model was the approximation of the sensitive interface as a concave bend. The developed model was validated by experimental results.

Finally, the development of optical fiber sensors is not limited to the use of only one transduction method; they can involve both methods [49,50], or the combination with other techniques, such as surface plasmon resonance (SPR) [51,52].

The major disadvantage of sensors based on intensity variations is related with the stability of the emission sources, which induces errors in the measurements and limited the resolution. However, to overcome this drawback, self-referencing techniques can be used. The simplest method is to split the optical signal in two optical fibers, being one the reference beam. Another method is the use of several emission sources, wherein only one wavelength is attenuated [53].

### 3.2. Path Difference Based Sensors

Another technique used in the design of optical fiber sensors is based on changes in the phase of a modulated optical signal due to different optical fiber length and hence different transit times [30]. The optical fiber length can be changed with temperature, bending and longitudinal deformation.

Path difference based sensors can be obtained comparing the phase of an optical signal that propagates along two different paths, being one the reference fiber. Considering a sinusoidally intensity modulated optical signal, if one of the fibers is lengthened the phase difference (*φ*_1_ − *φ*_2_) between both optical signals changes [30,54,55]. This change in the phase difference can be obtained in the electric domain using a phase comparator. The difference in length among the two fibers (ΔL) can be calculated from the phase difference shift (Δ(*φ*_1_ − *φ*_2_)) by the following expression [30,55]:

(1) $$\Delta L=\frac{c_{0}}{n_{co}f_{m}}\frac{1}{360}\Delta (\varphi_{1}-\varphi_{2})$$

where *c*_0_ is the speed of light in vacuum, *n_co_* is the core refractive index and *f_m_* is the modulation frequency. The used modulation frequency will depend on the structural elements under test and is upperly limited by the finite bandwidth of the fiber, the transmitter and the receiver.

### 3.3. Other Sensing Techniques

The most common component for wavelength detection based sensing is the FBG. Although most FBGs referred in the literature were inscribed in GOFs, the investigation of FBG inscription in POF is under investigation since the maximum elongation of silica is around 3% [56]. The first FBG in SI-POF was produced in 1999 [57]. The photopolymerization is the main mechanism that regulates the Bragg gratings inscription process. In order to enhance the photosensitivity and reduce ultraviolet (UV) degradation, trans-4-stilbenemethanol was used as an active dopant in PMMA [58]. Other works were published reporting the inscription of FBGs with distinct λ_B_ [59] and the use of different dopants [60]. The polymers and silica properties clearly differ from each other and thus provide additional benefits, essentially due the Young's modulus being about twenty five times less than that of silica. For a FBG with a λ_B_ of 1,570 nm, 28 dB-transmission loss and a half width of 0.5 nm, a sensitivity of 1.48 pm/με and 55 pm/°C were achieved for deformation and temperature, respectively [61,62].

POF development has also focused on microstructured designs, with considerable progress in the manufacture of mPOF [63]. The geometry of this type of fiber provides different properties compared to a step-index fiber, such as an endlessly single-mode, air-guiding operation and the ability to expose the electric field of the guided mode to substances contained within the holes. Several applications for mPOFs in mechanical sensing, fluids detection and spectroscopy using evanescent-field interaction can be found in [64]. The first FBG inscribed onto mPOF was obtained by Dobb *et al.* [65], using a HeCd laser at 325 nm. In 2008, Webb *et al.* [66] presented the first FBG written in POF fabricated from TOPAS, a cyclic olefin copolymer that has a temperature response similar to PMMA but a lower water affinity. In 2010, the first FBG in PMMA mPOF with λ_B_ in the 800 nm region was developed by Jonhson *et al.* [67]. In this spectral band, the fiber has an attenuation coefficient of 10 dB/m that is lower than the typical 1 dB/cm of the C-Band.

On the other hand, when high precision is required, interferometry can be used. The development of SM POF sensors is presenting a significant increase due to the fabrication of this fiber with low attenuation coefficients [11]. However, currently Paradigm Optics is the sole supplier. All the studies that will be described in this section used this optical fiber.

The most frequent interferometer arrangement using POF is the Mach-Zehnder configuration. Kiesel *et al.* [68] presented a configuration for the measurement of the phase shift in a PMMA SM optical fiber as a function of nominal strain. A phase sensitivity of 1.39 × 10^7^ rad.m^−1^ was measured with 15.8% nominal strain, a range much larger than the yield point. In a sequential study [69], the nonlinear phase sensitivity was evaluated (3.1 × 10^6^ rad.m^−1^) and the mechanical and photoelastic nonlinear coefficients were also calculated, being 11.4 and 16 respectively. In order to validate a SM-POF sensor and an interrogator for large strain measurements, this Mach-Zehnder interferometer was rearranged to allow an automated measurement of the phase shift [70]. A Mach-Zehnder interferometer based on the same approach was used to measure the phase shift induced by an ultrasonic wave emitted by a calibrated piezoelectric transducer on part of the optical fiber under test and immersed in water [71]. The detection sensitivity of the POF based interferometers was greater one order of magnitude when compared to the silica based configuration. A similar experimental setup was used to develop a photoacoustic microscope [72].

The Fabry-Perot interferometer doesn't require a reference fiber. In this configuration the fiber has two partially reflective mirrors. The partially transmitting mirrors cause the light to travel multiple paths inside the cavity, magnifying the phase difference and doubling the sensitivity. The first interferometric sensing cavities written in PMMA was obtained by using a Fabry-Perot cavity [73].

## 4. Applications

A review of some of the most important applications of POF based sensors is presented in this section. Structural health monitoring, biomedicine, environmental and biochemical areas will be discussed.

### 4.1. Structural Health Monitoring

In the literature, there are a considerable number of papers dedicated to the exploration of POFs as strain sensing elements. Most are devoted to structural health monitoring [11,74,75]. Kuang *et al.* [31] presented a study where the performance of a sensor based on the separation between two longitudinal POFs was evaluated in quasi-static tests. The sensor was placed on an aluminum sample, showing a high linearity between the deformation of the sample and the transmitted power. Dynamic tests on a cantilever and a load impact experiment were also carried out to evaluate the system's ability to detect the vibration modes of the structure. Further work focused on the integration in geotextiles, enabling the measurement of deformations around 5% in compression tests and up to 40% in tensile tests [76]. Using a similar setup, Sun and Oyadiji [77] studied the dependence between the cantilever material and the sensor performance. The authors concluded that, in quasi-static tests, the use of soft materials (e.g., rubber) resulted in a greater sensitivity. However, materials with a higher stiffness, such as polytetrafluoroethylene (PTFE), provided quicker response times in dynamic test (1 kHz). Several studies for distributed strain measurements in geotextile have been also published. One of the methods is based on OTDR, a time-resolved technique for the detection of backscatter light intensity of single pulses injected into the fiber [24–26]. Within the scope of the FP6-NMP funded Polyfunctional Textiles against Natural Hazards (POLYTECH) project, geotextiles were applied in the monitoring of sediment movement on slopes and geotechnical and civil structures [24]. Studies were also performed in PMMA and PF-GI-POF based systems [26]. The noise level typically associated to the OTDR and the resultant decrease in dynamic range of operation and spatial resolution led the investigators from the Federal Institute of Materials Research and Testing, Berlin (BAM) to compare the performance of the OTDR to a OFDR. This work pioneered the exploration of this technique for distributed strain sensing. The results showed a significant improvement in response time and in the dynamic range of operation [27]. Using OFDR in PF-GI-POF, another innovation was achieved by the same team, resulting in an increase to 500 m of the measurement length, with a resolution in the centimeters range [28].

In order to monitor the development of cracks, fissures and small displacements in concrete structures, Perrone *et al.* [78] developed a sensor with sub-millimeter resolution based on the phase difference of the consequent deformations imposed on a structurally integrated POF. With reference to distance measurements, Casalicchio *et al.* [79] reported a low-cost system whose experimental setup was designed in order to compensate errors associated to the different reflectivity of the possible targets. The system consisted in two fibers, one used for the emission and both for the collection of the signal reflected by the surface. The same operating principle was applied in the development of accelerometers [80,81].

A sensor based on microbending was designed to monitor the cure process of cement. The fiber was placed in the plastic phase of the material and, as the cure evolved, the cement/fiber system became deformed. The additional attenuation in the transmitted power was correlated with the curing of the cement [82]. Recently, André *et al.* [83] proposed a method to determine the content of concrete' water during its curing process. The sensing technique was based on the scattering of the optical signal in grooves performed on the plastic fibers. With this solution, the curing process phases could be perfectly distinguished. The high sensitivity provided, the facility of implementation and the low-cost associated were the main advantages of the presented method. Although not associated with SHM, Bilro *et al.* [84] presented a compact, very simple and low-cost solution for cure monitoring of several materials. They used a side-polished POF system, being the proposed sensor based on the changes of the optical properties of the material during the cure process, namely the refractive index. Thus, the reflection and transmission light at the interface of the two dielectric media was traduced by Fresnel's equations.

**Figure 6.**
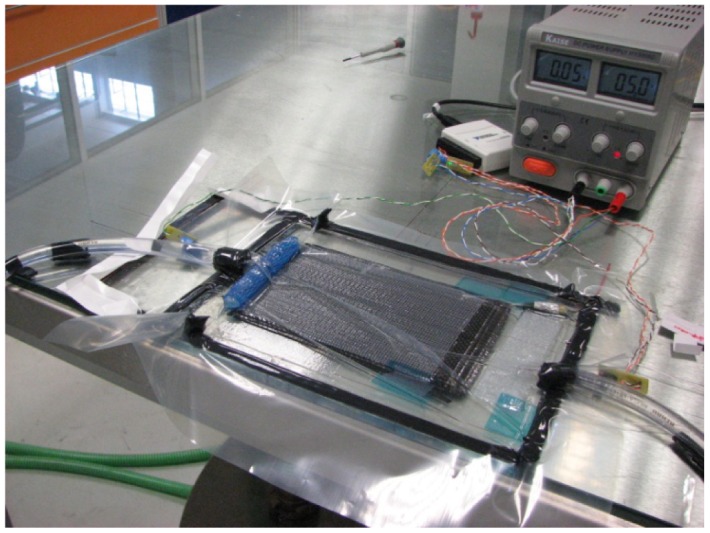
Vacuum infusion process setup for the production of carbon fiber reinforced composite structures [86].

A recent area where POF sensors have been applied is in the development of smart structures. Cortes *et al.* [85] presented a study in which either GOF-FBG and POF sensors were introduced into a glass fiber laminated composite structure and into a shape memory Ni-Ti alloy. The POF sensor was based on the measurement of the displacement of two cleaved fiber surfaces contained within the tube. The sensors were introduced to monitor the deflection of the laminate during the activation phase. Both types of sensors were also integrated into carbon fiber reinforced composite structures. However, in this study the principle of the POF sensor relied on intensity variation in a side-polished fiber. This integration allowed a real time monitoring of the production process (shown in Figure 6), including the progression of resin cure over time and the determination of the infusion rate [86].

The previously referenced works are relevant for the development of composite structures used in civil engineering, aeronautics and aerospace, in order to assess the reliability, durability and safety. Therefore, it is important to develop lightweight, compact and low cost systems that allow the control and the detection of possible structural damage. As an example, a system for monitoring the structural integrity of a wing of an airplane was developed, resulting from a collaboration between the University of the Basque Country, the Aviation Technology Center and the POF-AC. This device contained an elongation sensor based on two POF. Several tests were carried out (deflection point, step by step and cyclical movements) and it was concluded that the FBG-GOF and the POF sensor had a similar performance [30,54,55].

Regarding aerospace engineering instrumentation, Ge *et al.* [87] investigated the properties of several POFs (polystyrene—PS, polycarbonate—PC and PMMA) and their performance under exposure to high doses of gamma radiation. The authors concluded that the damage is dependent of the induced radiation wavelength.

For 1,000 Gy, similar decreases of the transmitted power were observed in the entire visible electromagnetic spectrum. On the other hand, for 5,000 Gy a sharp decrease in the wavelength range 400 nm–500 nm was noticed. For PMMA based POF there was a minimum in the range 550 nm–650 nm and for PS based POF a similar result was obtained in the range 500 nm–700 nm. In the case of these gamma radiation dose levels, the recovery process of the structural damage induced to the fibers was slow or irreversible.

### 4.2. Medicine

The medical field is another important application of POF based sensors. Witt *et al.* [88] applied the OTDR technique in the development of POF-integrated textiles for monitoring respiratory movements in anesthetized subject, during a magnetic resonance (MR) imaging. Yoo *et al.* [89] presented two different non-invasive respiration sensors. One consisted in a nasal-cavity attached sensor that could measure temperature variations of air-flow based on color changes of a thermochromic pigment. The other device was an abdominal size sensor, comprising a sensing branch of PMMA tubes, a mirror and a spring.

Morisawa *et al.* [90] developed a system based on five humidity POF sensors for the recognition of devoiced vowels. Through the moisture pattern formed in the pronunciation, the system had a recognition rate of 93%. In order to design medical devices that enabled a simultaneous electrical and optical stimulation, Kim *et al.* [91] investigated the possibility of adhere gold thin film to PMMA and PF fibers and so provide electrical conductivity to the POF. The results showed that the combination of ion sputtering, the introduction of roughness and the use of a Ti layer substantially improved the adherence of the gold thin films to the substrates. In the photodynamic therapy field, Jeoung *et al.* [92] studied the possibility of producing power splitters based on tapered POF with N inputs and N outputs (N up to 60 units).

In physical medicine and rehabilitation, namely in the measurement of joint movement, optical fibers have been attracting attention since 1988. This first sensor consisted of two optical fibers with their ends aligned and placed in a deformable tube [93]. This system was intended to be laterally applied into the phalangeal articulation with the fixed sensor attached to the proximal zone of the joint and the mobile sensor on a guide in the distal area. Preliminary results revealed that such setup was feasible for a monitoring joint angular motion sensor. Two other studies showed an angle sensor whose operating principle was based on the intensity variation of the optical signal propagated through the fiber when subjected to macrobending [94,95]. Despite the positive results, these works had some limitations. There were sources of error that made the system unstable and inaccurate. These errors could be due to undesirable bends, fluctuations in the optical power and hysteresis. Thus, to solve these problems, new approaches appeared. In order to develop a system to assist handicapped people to move in a wheelchair, Lee *et al.* [96] presented a system constituted by two side-polished optical fibers placed on the shoulder of a user. The polished side increased the sensitivity to bending and allowed the measurement of concavity. If an optical fiber end presents an asymmetry, the optical power detected by a photodetector depends on the angle of curvature. Despite the results obtained by Lee *et al.*, further clarification regarding the relationship between detected intensity and the photodetector bias current, the cutting angle and bending angle was proposed, as well as the development of a more sensitive method of detection [97].

**Figure 7.**
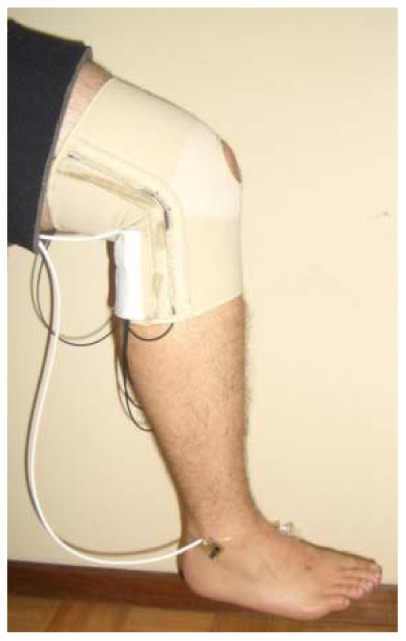
Plastic optical sensor integrated in a commercial knee support.

Recently, Bilro *et al.* [98] presented a wireless and wearable system designed to evaluate quantitatively the human gait, allowing knee sagittal motion monitoring over long distances and periods with a portable, friendly operation and low-cost package. The core of the system was the measurement of transmittance changes when a side-polished POF was bent (Figure 7).

In this condition the critical angle for total reflection will decrease or increase as the bending is convex or concave, respectively. The system was tested in order to assess inter-day and inter-subject reliability. Results revealed that the prototype was reliable, allowed a one-time calibration and was suitable in the diagnosis and rehabilitation of knee injuries or to monitor the performance of high competition athletes. The same authors reported two more optical monitoring joint angle devices, with the ability of real-time assessment of the elbow and ankle movements [99]. The three monitoring devices (knee, elbow and ankle) were tested in different selected tasks and its performance evaluated. The ability of these systems to deliver quantitative data about different activities was proved, even in one of the most frequent daily activity, sit-to-stand, by the ankle optical system. The knee monitoring system was able to detect some slightly gait deviations when walking with footwear or running and the elbow device allows the determination of the extension angular velocity in a throw.

### 4.3. Environment

The cost of commercially available sensor systems for continuous monitoring of environmental parameters is a constraining factor. Hence, this has been another area of growing interest in polymeric fiber solutions. For instance, using a sensing technique based on chromatic indicators that change their color in the presence of certain species, Yokota *et al.* [100] developed a system able to determine six different nutrients in soil samples. The configuration also involved the use of multiple LED whose wavelengths were chosen with regard to the absorption band of the chemical reagents when entering into contact with nutrients. Kuang *et al.* [101] implemented a network level sensor, where the power loss which propagated in the fiber was related with the liquid contact. A network of five sensors was integrated to detect the rising water level in a basin. Also in this issue, Aiestaran *et al.* [102] proposed a sensor to determine the flow rate and turbidity of a low-viscosity fluid, in this case water, as it passed through a pipe. The sensor was integrated in a rotating propeller placed in a pipe, perpendicular to the liquid movement. In the same work, a PMMA POF was used for turbidity measurements using different colour dyes.

**Figure 8.**
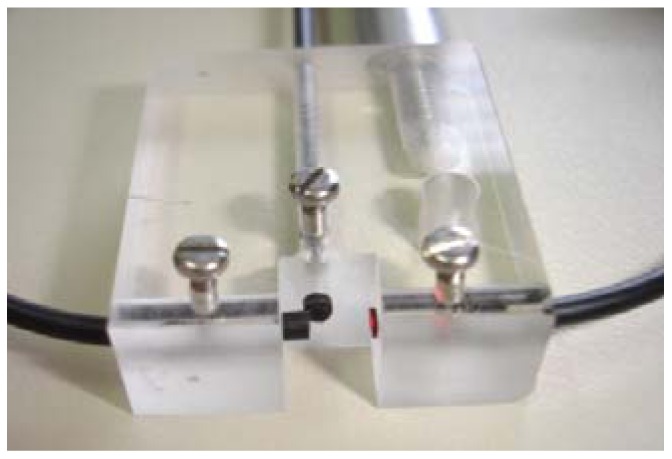
Low-cost plastic optical turbidity sensor.

Regarding turbidity sensors, there are some studies for marine applications [103] and wine industry applications [104]. These particular studies were focused in the determination of low suspended sediment concentrations, typically 2–4 g·L^−1^. On the other hand, Campbell *et al.* [105] presented a sensor designed for measuring high sediment concentrations, up to 10 g·L^−1^. The system was created to perform measurements using a linear fiber bundle design. However, due to the size of the suspended particles, the authors didn't solve the variability of the optical signal. Later, Postolache *et al.* [106] developed a system with four optical fibers, two used as emitters and two as receivers. Despite the apparent complexity of the system, its capability was not optimized, since both transmitted and scattered signals were used for the determination of only one parameter, in this case the concentration of suspended particles. In addition, the sensor performance was not tested in real samples. The system proposed by Bilro *et al.* [107] was also based on the transmission and nephelometric method, similar to the one presented by Garcia *et al.* [104]. Transmitted and scattered output signals were characterized and evaluated for three configurations, using a large range of clay concentration. The configuration selected was the one that presented the best balance between the sensitivity of the sensor and its capacity to operate with suspended particles of large dimensions that are a characteristic in soil erosion studies (see Figure 8). Comparing to OBS-3+, a standard commercial system, a good correlation was obtained. Nevertheless, authors pointed that with the developed system the operation mode was easier, because it was small sized, cost-effective and the output didn't depend on the setup. Later, a study of the sensor performance was presented [108]. This was tested with artificially-created samples, with a wide range of concentration of three distinct materials, *i.e.*, white flour, red clay and black ashes. Results showed that direct light loss proved to be a good indicator of the sediment concentrations of the artificial samples and, also, the scattered-light signal indicated potential as a complementary signal for the identification of the reflectivity of the particles. A second test was accomplished to determine the sensor's sensitivity based on six particle size classes of ashes. Results suggested that the concentration misreading due to particle diameter could be corrected using output signals variability.

Montero *et al.* [109] proposed an intensity based sensor for liquid detection. The operation principle was related with the bending losses of a partially polished POF coupler. The coupling ratio was dependent of the different refractive indices that surrounded the optical fiber. Compared with other techniques, the advantage of the presented sensor was its self-referencing system which avoided the addition of other elements to eliminate undesirable intensity perturbations on the measurements. The developed device can be used in harsh environmental, where liquid level measurements are required.

### 4.4. Biological and Chemical

A variety of biological and chemical species can be detected using POF technology-based sensors. Sometimes, the fiber itself can play an active role by acting as a sensing probe. The activation can be accomplished by replacing the original cladding material or coating the end of the fiber with a chemical sensitive material. In the literature, a wide number of sensors make use of this technique. For instance, using the reflection method, Goicoechea *et al.* [110] developed an optical fiber pH sensor based on the indicator neutral red. The results showed that a multi-layered structure composed of an acrylic acid polymer and the indicator was the optimal method for making the nanostructured layer containing the pH sensitive element. This solution had a dynamic operating range of 2.5 dB at a pH between 3 and 9. Rovati *et al.* [111] developed a sensor for the same purpose using a drop of sol-gel hybrid material containing phenol red deposited onto the tip of a large core POF. When there was a change in the dye colour, the light collected by the fiber varied. The working range of the proposed pH sensor was 5–8.

Nagata *et al.* [112] reported a methanol and ethanol sensor, wherein the new deposited cladding had a refractive index slightly above the refractive index of the core. In the presence of alcohol, the structural properties of the cladding were changed and the refractive index decreased to values below the refractive index of the core, enhancing the power output. In the last years, the interest on these sensors has grown; ethanol and methanol are being used as an energy source and also due of its toxicity and flammability. The same principle of operation was applied by Fujii *et al.* [113] in the development of a toluene sensor (aromatic toxic compound) using high density polyethylene as modified cladding material. The concentration of toluene was correlated with the output signal. A minimum detection limit of 1% v/v and a response time of about 1 second were obtained.

With the growing interest in preserving the cultural heritage, Angeline *et al.* [114] developed an etched plastic fiber sensor for *in-situ* monitoring of H_2_S, which is the chemical compound responsible for the tarnishing of silver artifacts. The sensing principle of the proposed sensor was based on the variation of the guided light intensity due to chemical reaction between a thin silver layer deposited onto the plastic fiber core and the sulfite components to be detected. The sensor was tested in laboratorial environment and the results revealed that H_2_S concentrations as low as few part-per-billion (ppb) could be detected.

Varghese *et al.* [53] developed a side-polished POF to determine the concentration of silica present in water. The detection scheme was based on two wavelengths; one corresponding to the absorption maximum of the chemical species (815 nm) and the other for self-referencing the system (640 nm). An electro-active chip was developed combining lateral polishing and spectroscopic methods. The POF was polished and coated with a thin film of indium tin oxide, acting as an electrode. Due to the presence of surface-confined redox species, electrochemical properties variations were detected [50].

In order to detect *Legionella pneumophila*, Lin *et al.* [115] proposed an immunosensor based on a side-polished optical fiber. The sensing principle relied in the SPR technique. The fiber was side-polished down to closing half the core and coated with a 37 nm gold thin film by DC sputter. The sensitivity of the SPR fiber for the *Legionella pneumophila* was confirmed with the detection limit of 10^1^ CFU/mL and the detection range of 10^1^–10^3^ CFU/mL.

Leung *et al.* [116] presented also a biosensor based on a tapered fiber coated with gold housing in a flow cell. For the detection of single-stranded DNA, authors showed that it was feasible to directly detect the hybridization of this DNA to its complementary strand immobilized on the sensor surface. Detection was performed under flow conditions for nonspecific binding to sensor surface and eliminating optical transmission changes due to mechanical movements. Additionally, it allowed instantaneous switching of samples, when needed. The sensor also showed selectivity against a single nucleotide mismatch. A similar study for the continuous detection of various concentrations of bovine serum albumin was presented. They also monitored a target serum in the presence of a contaminating protein (ovalbumin) [117]. Concerning biosensors, Beres *et al.* [118] applied a chemical treatment to several POF tapers in order to allow the attachment of specific antibodies. The cells, when linked to their antibodies, induced a variation in the refractive index close to the surface of the taper. Results showed that this approach enabled the detection of *E. coli*, yeast cells and lamb blood erythrocytes.

Dikovska *et al.* [119] proposed an ammonia fiber optic sensor using a zinc oxide nanostructure grown on the side-polished fiber section. The sensor element operation principle was based on a distributed coupling between the fiber mode and the metal oxide planar waveguide mode (SPR phenomenon). The sensor performance was evaluated under ammonia gas at room temperature. The concentration limit achieved was 50 ppm.

Chu *et al.* [120] used a ruthenium complex ion to build an oxygen sensor based on fluorescent methods. The indicator was placed as a film, using the sol-gel method. Platinum (Pt) complexes are also used to detect oxygen by fluorescent techniques. These dye complexes are easily excited using compact and low-cost LED light sources. Furthermore, the phosphorescence wavelengths of Pt and ruthenium (Ru) are well separated from the excitation LED wavelength and hence the influence of the excitation light source can be easily eliminated. One source of error in these systems is the temperature, since the diffusion of oxygen depends on this parameter. To compensate the temperature-induced variations in the luminescence intensity, an appropriate calibration factor is needed [121]. Ganesh and Radhakrishnan [122] coated the sensitive area with black silicone, avoiding interferences due to changes in the environmental conditions, namely refractive index, turbidity, reflectivity, ambient light, and background fluorescence in sediments and biofilms. The oxygen determination by measuring the fluorescence lifetime of metal organic Ru complexes can be an indirect method for other chemical species. Scully *et al.* [123] developed a glucose sensor based on the oxygen consumption of the enzymatic conversion of glucose to gluconic acid. Rivera *et al.* [124] proposed a sensor for lead ions (Pb^2+^). They applied fluorescence methods and used a membrane of polyvinylchloride (PVC) sensitized to respond to the presence this metal. A detection limit of 7 × 10^−6^ M was obtained.

## 5. Conclusions

This paper has presented a review of recent research and developments of POF sensor technology, with special focus on intensity variation schemes and low cost solutions. They can be considered a valuable alternative to traditional technology. The market is still demanding low-cost solutions where POF can play a relevant role. On the other hand, the different mechanical and thermal properties of polymers allow new possibilities for the development of multiparameter sensors, new modulation schemes and embedded systems for several target applications e.g., textiles, composite and concrete integration.

The main thrust of this technology should be focused in reliability and standardization. Research should also be dedicated to the development of new POF materials, geometries and interrogation techniques to overcome the high attenuation coefficients, the low temperature of operation and to increase chemical resistance to organic solvents. The improvement of the connectorization processes should also be addressed. In conclusion, it is expected that POF-based sensors will continue to be a very active research topic in the near future.

## Figures and Tables

**Table 1.** Plastic optical fiber suppliers.

**Step Index Fibers (PMMA)**	**Graded Index (PMMA)**	**Graded Index (PF)**
Asahi Chemical (Luminous)	FiberFin	Asahi Glass
Luceat	Fuji Photo Film Co	Chromis Fiberoptics
Mitsubishi International Corp.	Nuvitech	
Nuvitech	Optimedia	
Toray Industries, Inc.	COMOSS	
COMOSS		
Avago Technologies		
